# An Eustachian Tube Neuroendocrine Carcinoma: A Previously Undescribed Entity and Review of the Literature

**DOI:** 10.1155/2016/4643615

**Published:** 2016-06-27

**Authors:** Gavin J. le Nobel, Vincent Y. Lin, Vladimir Iakovlev, John M. Lee

**Affiliations:** ^1^Department of Otolaryngology, Head and Neck Surgery, University of Toronto, Toronto, ON, Canada M5G 2N2; ^2^Sunnybrook Health Sciences Center, Toronto, ON, Canada M4N 3M5; ^3^St. Michael's Hospital, Toronto, ON, Canada M5B 1W8

## Abstract

Primary sinonasal and middle ear neuroendocrine carcinomas are rare malignancies of the head and neck. Owing to the rarity of these tumors, the clinical behavior and optimal management of these tumors are not well defined. We present a case of an incidentally discovered sinonasal neuroendocrine carcinoma that was found to originate from the Eustachian tube, which has not previously been described in the literature. This patient was treated with primary surgical resection using a combination of transnasal and transaural approaches and achieved an incomplete resection. Follow-up imaging demonstrated continued tumor growth in the Eustachian tube as well as a new growth in the ipsilateral cerebellopontine angle and findings suspicious of perineural invasion. However, the tumor exhibited a benign growth pattern and despite continued growth the patient did not receive additional treatment and he remains asymptomatic 35 months following his original surgery.

## 1. Introduction

Primary sinonasal neuroendocrine tumors are rare [1]. Four histologic phenotypes of sinonasal neuroendocrine tumors exist: esthesioneuroblastoma, sinonasal undifferentiated carcinoma, small-cell undifferentiated carcinoma, and neuroendocrine carcinoma (NEC).

NECs are further classified as well-differentiated (typical carcinoids), moderately differentiated (atypical carcinoids), and poorly differentiated (small and non-small-cell types) [2, 3]. Owing to their rarity, the clinical behavior and optimal management of sinonasal NECs are not well established. To complicate matters further, many early studies of NEC have included a range of sinonasal neuroectodermal tumors including olfactory neuroblastoma, small-cell NEC, and sinonasal undifferentiated carcinoma. We present a case of NEC arising from the Eustachian tube, which has not previously been reported in the literature, and review the literature regarding sinonasal NECs.

## 2. Case Report

**Figure 1:**
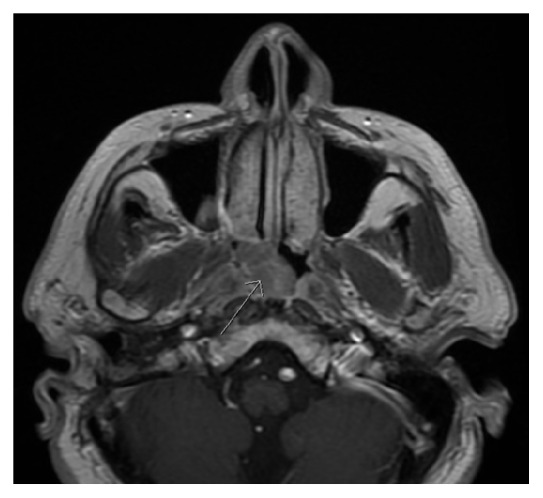
T1 weighted axial MRI demonstrating incidentally found polypoid, exophytic mass in the right nasopharynx.

A 68-year-old man presented with an incidentally discovered 2 × 1.6 cm polypoid, exophytic mass in his right posterior nasopharynx found on imaging done as part of a stroke workup (Figure 1). He had a longstanding history of nasal obstruction and discharge but was otherwise asymptomatic. Past medical history included type II diabetes, hypertension, stroke, cardiovascular disease, benign prostatic hypertrophy, and recurrent right sided otitis media treated with mastoidectomy in 1971.

**Figure 2:**
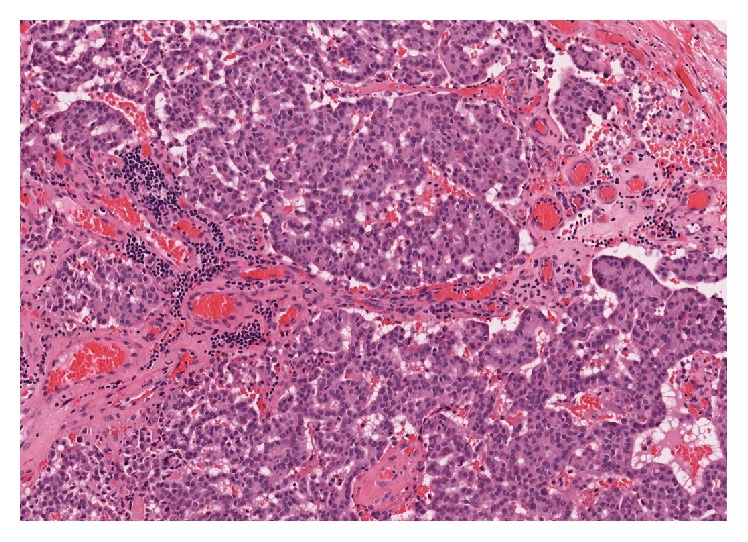
Hematoxylin and eosin stain of tissue removed from left nasopharynx at 20x demonstrating nest of tumor infiltrating soft tissues.

**Figure 3:**
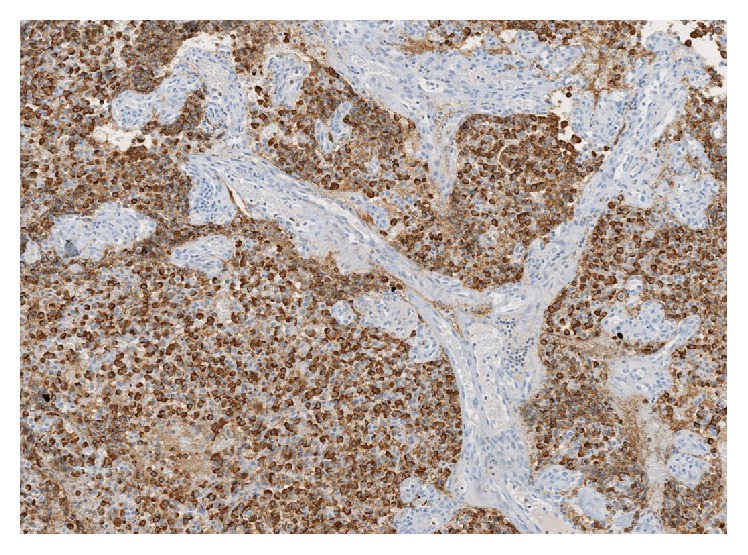
Chromogranin stain of tissue removed from left nasopharynx at 20x confirming neuroendocrine origin of tissue.

Two months later, clinical examination revealed a violaceous, vascular, benign-appearing lesion pedicled to the right Eustachian tube orifice. Aural examination revealed evidence of previous mastoid surgery but no other abnormalities. Owing to concern of aspiration, biopsy was not initially attempted and he was referred for single stage excision. The lesion was removed endoscopically; however, the origin of the tumor could not be easily resected transnasally and given the mass' benign appearance, this was not aggressively pursued. Consequently, a small amount of the tumor pedicle was left. Pathology revealed moderately differentiated NEC (Figures 2 and 3) and subsequent workup found that chromogranin A and urine 5HIAA levels were normal.

**Figure 4:**
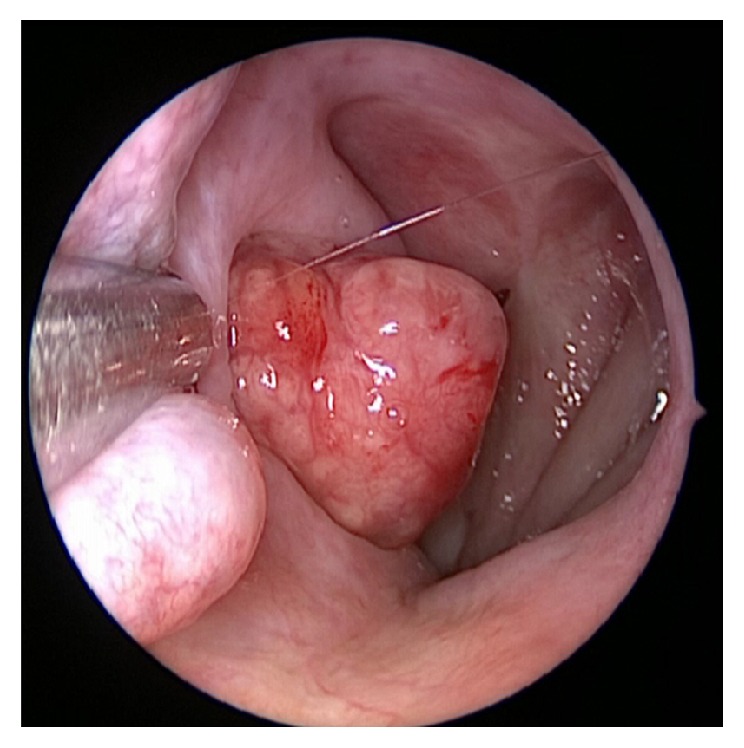
Recurrent tumor shown at the time of his second endonasal surgery.

Examination two months following demonstrated no evidence of recurrence. Due to the unusual location of this tumor and to rule out another primary, an octreotide scan as well as CT scans of the head, neck, chest, abdomen, and pelvis was done. These demonstrated no other focus of disease; however, they confirmed residual disease in the Eustachian tube. With residual tumor, it was felt that there was, as yet, no role for chemotherapy. Repeat examination five months following surgery demonstrated a 5 mm nodule of tumor at the introitus of the Eustachian tube and he underwent transnasal resection of the residual mass 1 year following his original surgery (Figure 4). Most of the tumor was resected en bloc and dissection was carried to the limits of the instruments, at the bony-cartilaginous junction of the Eustachian tube. However, the tumor pedicle continued further up into the middle ear space. Six weeks earlier, a vascular mass in the right external auditory canal had been identified and a biopsy was taken. Pathologies demonstrated moderately differentiated NEC.

Four months following this surgery, he underwent a microscopic transmastoid right subtotal petrosectomy with microdebridement of his tumor. Tumor was found in the epitympanum and sinus tympani and partially covered the anterior wall and completely covered the medial wall of the middle ear. Most of the tumor in the middle ear space was removed and dissection was carried to the limits of instrumentation. There was, however, some residual tumor in the Eustachian tube and along the stapes footplate that could not be removed. Pathology demonstrated the aforementioned NEC.

**Figure 5:**
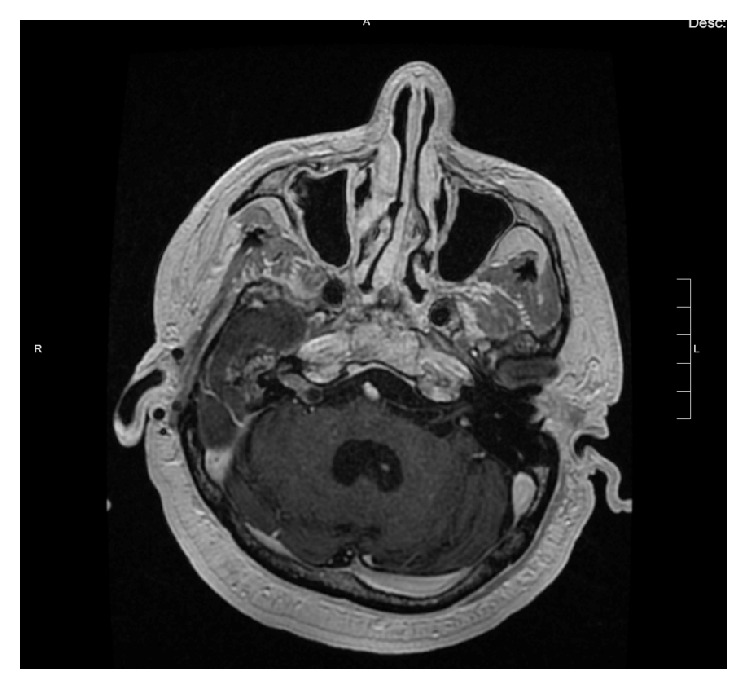
T1 weighted axial MRI demonstrating tumor growth in the cerebellopontine angle.

Follow-up MRI done three and eight months following this surgery demonstrated an area of enhancement anterior to the right Eustachian tube that increased from 14 × 6 mm to 14 mm × 8 mm in the intervening time. The second MRI also demonstrated a 17 × 3 mm enhancing lesion in the right internal auditory canal with an extracanalicular cerebellopontine angle component in addition to perineural invasion (Figure 5). Three weeks following this second MRI, he was started on sandostatin; however, no further attempts at surgical resection were made. One week following this second MRI, he developed sudden onset and dense facial nerve paralysis which did recover over the subsequent 5 months. He remains, otherwise, asymptomatic 35 months after his initial surgery.

## 3. Discussion

Sinonasal NEC was first proposed as a distinct entity in 1982 when it was appreciated that certain small-cell tumors behaved distinctly from other olfactory neuroblastomas [4]. These tumors behaved similar to less differentiated neuroendocrine tumors [4, 5]. Sinonasal NECs are thought to arise from submucosal glands, in particular Bowman's glands, and have been defined as a malignant neoplasm with evidence of neurosecretory granules but, in contradistinction to olfactory neuroblastoma, lacking evidence of a neurofibrillary background by light microscopy [4, 5].

### 3.1. Presentation

Average age of presentation of sinonasal NECs is 50 years [2–10]. More cases have been reported in males; however, a statistically significant gender predilection for sinonasal NEC has not been demonstrated.

The most frequent presenting symptoms are nasal obstruction and epistaxis. Two case series summarize the most frequent presenting complaints for sinonasal NECs (Table 1) [2, 4]. Patients with NEC have also presented with proptosis, extraocular muscle palsy, and facial pain and numbness [3, 6, 7, 10]. Mean time elapsed between symptom onset and diagnosis is 10 months [2]. Our patient presented incidentally and was asymptomatic.

Most often, the precise origin of sinonasal NECs cannot be ascertained and, in one series, the tumor origin could only be identified in 30% of cases [4]. However, most sinonasal NECs originate from the superior nasal cavity, the superior turbinate, and the ethmoid sinuses but have also reportedly originated in the middle turbinates, the remainder of the nasal cavity, and the maxillary and sphenoid sinuses [3–5, 8, 10]. No reported cases of NEC originating from the Eustachian tube exist. Consequently, the possibility that this tumor was metastatic in origin was investigated and two octreotide scans did not corroborate this.

The unique site of origin of this tumor implies surgical challenges that are not encountered with previously reported sinonasal and middle ear NECs and distinguishes this case from previously reported NECs of sinonasal or middle ear origin. NECs of sinonasal origins may potentially be addressed using endoscopic skull base resections while middle ear tumors may be resected using microscopic techniques, possibly including lateral temporal bone resection. By contrast, the anatomic considerations of resecting a tumor originating from the Eustachian tube are unique and would require a much more invasive middle fossa approach, if an aggressive surgical resection was desired.

Most patients with sinonasal NEC present with advanced local disease but rarely with locoregional or distant metastases. One series reported that nearly 80% of patients presented with moderately advanced or very advanced local disease [2]. Only 10% presented with lymph node involvement and none presented with distant metastases [2]. Studies have not demonstrated differences in tumor size, nodal involvement, or distant metastases between well-differentiated, moderately differentiated, and poorly differentiated sinonasal neuroendocrine carcinomas at presentation. In our patient, the NEC was at stage 1 at presentation with a T1 primary tumor and without evidence of metastases.

### 3.2. Treatment

Owing to limited published data regarding the behavior and treatment of sinonasal NEC, treatment decisions have often been adapted from NECs of other anatomical sites, such as lung and larynx [2]. Treatment varies due to lack of consensus; however, patients have been treated with multimodal approaches involving surgery, radiation, and chemotherapy.

Neoadjuvant treatment of sinonasal NEC varies. In one series, 15% of patients received preoperative radiation while in another, none received preoperative radiation [2, 4]. By contrast, in the first series, none received neoadjuvant chemotherapy while in the another, 35% received neoadjuvant chemotherapy [2, 4]. Our patient was not treated neoadjuvantly.

Most patients receive surgery as part of their treatment, with reported rates between 75% and 80% (Table 2) [2, 4]. Surgical resection, however, is frequently incomplete with one series reporting a rate of incomplete resection of 40% [4]. In our patient, complete tumor resection could not be achieved with minimally invasive approaches and given the apparently indolent nature of the malignancy, a more invasive middle cranial fossa approach was not considered.

Patients treated initially with surgery with or without neoadjuvant chemotherapy frequently receive adjuvant chemotherapy and/or radiation, most commonly adjuvant radiation [2, 4]. Following surgical resection with involved margins, 50% received adjuvant chemoradiation and 33% received adjuvant radiation [2]. In our case, although surgical resection was incomplete, no adjuvant treatment was given.

### 3.3. Prognosis

Current combined modality treatment of NECs has led to good disease control and survival rates [3]. In one series, following successful primary treatment, survival was reported to be 100%, 88%, and 77% at 5, 7, and 10 years, respectively [4]. Furthermore, within the follow-up period (range 1–19; median 5.5 years) only 18% died as a result of their NEC [4]. Another series found poorer treatment results and tumor differentiation status was identified as a prognostic factor. Patients with moderately differentiated NEC had reported median disease-free and overall survival of 46 months and 107 months, respectively, while, for poorly differentiated NEC, median disease-free and overall survival were 11 and 47.5 months, respectively [2].

Recurrence following successful primary treatment of NEC is common, with locoregional more common than distant metastatic recurrence. Reported rates of recurrence for NEC are between 41% and 53% with approximately 75% developing locoregional and the remainder distant metastases [2, 4]. Mean reported elapsed time from treatment to recurrence is less than 4 years [2, 4]. Treatment of locoregional recurrences varies; however, the most frequent treatment approaches are outlined in Table 3.

In our patient, gross residual disease remained following initial treatment which has been associated with a poorer prognosis. One series reported that most patients with residual disease died of their disease within 18 months of initial treatment [2]. Our patient, however, developed a transient ipsilateral facial nerve paralysis which recovered. He is otherwise asymptomatic nearly three years after initial treatment.

## 4. Conclusion

This is the first reported case of sinonasal NEC originating from the Eustachian tube. Although NECs originating from the sinonasal tract and middle ear space have previously been reported, the anatomic confines and surgical considerations of this case of NEC which originates from the Eustachian tube render this a unique, previously unreported case. Pretreatment considerations should include tumor differentiation and surgical resectability. A multimodal treatment approach including surgery, chemotherapy, and radiation should be considered with these tumors.

## Figures and Tables

**Table 1:** Presenting clinical features of sinonasal neuroendocrine carcinomas.

Clinical feature	Silva et al., 1982 [4]	Likhacheva et al., 2011 [2]
Nasal obstruction/congestion	40%	50%
Epistaxis	20%	30%
Neck mass	10%	10%
Persistent sinusitis	5%	15%
Headache	—	15%
Anosmia	10%	—

**Table 2:** Treatment following primary surgery with or without neoadjuvant chemotherapy.

	Series	
	Silva et al., 1982 [4]	Likhacheva et al., 2011 [2]
None	23%	13%
Radiation	69%	47%
Chemotherapy	8%	7%
Concurrent chemoradiation	—	20%
Sequential chemoradiation	—	13%

**Table 3:** Treatment of locoregional recurrence of neuroendocrine carcinoma.

Silva et al., 1982 [4]		Likhacheva et al., 2011 [2]	
None	20%	None	0%
Surg	50%	Surg, Ad RT	40%
C	10%	Surg, Ad CRT	40%
Sequential C, RT	10%	NA C, RT, Ad C, Surg	20%
RT	10%		

Surg: surgery, C: chemotherapy, RT: radiation, CRT: chemoradiation, Ad: adjuvant, and NA: neoadjuvant.
